# Oral Anticoagulation Therapy: An Update on Usage and Costs in the Endemic COVID-19 Era

**DOI:** 10.3390/jcm14082591

**Published:** 2025-04-09

**Authors:** Emmanuel J. Favaloro, Leonardo Pasalic, Giuseppe Lippi

**Affiliations:** 1 Haematology, Sydney Centres for Thrombosis and Haemostasis, Institute of Clinical Pathology and Medical Research (ICPMR), NSW Health Pathology, Westmead Hospital, Westmead, NSW 2145, Australia; leonardo.pasalic@health.nsw.gov.au; 2 School of Dentistry and Medical Sciences, Faculty of Science and Health, Charles Sturt University, Wagga Wagga, NSW 2650, Australia; 3 School of Medical Sciences, Faculty of Medicine and Health, University of Sydney, Westmead Hospital, Westmead, NSW 2145, Australia; 4 Westmead Clinical School, University of Sydney, Westmead, NSW 2145, Australia; 5 Section of Clinical Biochemistry, University of Verona, 37129 Verona, Italy; giuseppe.lippi@univr.it

**Keywords:** direct oral anticoagulants, warfarin, vitamin K antagonist therapy, apixaban, rivaroxaban, dabigatran, anticoagulant therapy

## Abstract

**Background/Objectives**: Oral anticoagulant (OA) therapy (OAT) may be prescribed to patients for a variety of reasons, with several agent classes currently available as well as emerging. The classical oral anticoagulants are represented by vitamin K antagonists (VKAs), including warfarin, and the more modern alternatives comprise the direct oral anticoagulants (DOACs). We aimed to assess usage of OAs over time in Australia, especially focusing on the period of the coronavirus disease 2019 (COVID-19) pandemic and its transition to an endemic phase, to assess for any trends. **Methods**: Using data from the pharmaceutical benefits scheme (PBS), Medicare and other online sites, we specifically assessed for changes in OA prescription and cost patterns over the period 1992–2024, but focusing especially on the period 2020–2024 inclusive. **Results**: Apixaban is now the most prescribed OA in Australia. Costs of OAT prescriptions have steadily increased over the data capture period, reaching half a billion dollars in 2023. Interestingly, costs have started to fall, seemingly driven by the release of DOAC generics and PBS pricing adjustments. We could identify no clear signals related to COVID-19-related changes in prescription trends, contrary to previous reports in other locations. **Conclusions**: We provide Australian data on both OA usage as well as costs. Despite an ongoing trend to increasing use of DOACs over VKAs, we could not identify any specific COVID-19-related changes.

## 1. Introduction

Oral anticoagulant therapy may be prescribed to patients for a variety of reasons, with several agent classes currently available [1,2,3]. Several options exist including vitamin K antagonists (VKAs) and direct oral anticoagulants (or DOACs), as well as emerging novel oral anticoagulants (e.g., asundexian, milvexian) [3,4,5,6,7]. The classical oral anticoagulants, as represented by VKAs, include warfarin (acenocumarol, coumadin). VKAs remain commonly used for treatment of venous thromboembolism (VTE) [including deep vein thrombosis (DVT) and pulmonary embolism (PE)], stroke prophylaxis in atrial fibrillation (AF) and to reduce the risk of prosthetic heart valve thrombosis and thromboembolism. From the mid-2010s, a range of alternate oral anticoagulants (DOACs) became available, with the release of direct inhibitors of either thrombin (factor IIa) (dabigatran) or activated factor X (FXa) (e.g., rivaroxaban, apixaban, edoxaban). These newer agents have transformed the oral anticoagulant therapy landscape. These DOACs are sometimes alternatively called non-vitamin K antagonist oral anticoagulants’ (or NOACs).

We previously reported on the use and cost of oral anticoagulant therapy in Australia, in 2017 [7] and then in 2020 [8]. These reports showed the increasing adoption of the DOACs, as well as the increasing cost of oral anticoagulant medications. DOACs have a good safety and efficacy profile; they also do not need routine laboratory monitoring as they are prescribed in fixed doses for various clinical indications [9]. This is unlike the VKA agents, which need regular monitoring, most typically using the international normalised ratio (INR). In turn, the INR is a mathematical variation of a common laboratory test called the prothrombin time (PT) [10]. Since DOACs are approved for similar, as well as additional, indications to VKAs, they have surpassed the use of VKA therapy [7,8]. However, we also feel that increasing DOAC adoption also reflects a general trend to broadly increasing anticoagulation in otherwise anticoagulant naïve patients (e.g., improved recognition and higher prevalence of AF) [11]. Additional indications and more prolonged treatment (e.g., long-term secondary VTE prophylaxis) may also contribute to this pattern.

Our last update, published in 2020 [8], reflected data prior to the coronavirus disease 2019 (COVID-19) pandemic, which started in 2020, and is now considered an endemic condition. Although largely a respiratory infection, COVID-19 is also recognised to be a risk factor for thrombosis, due to activation of hemostasis and potential for a ‘cytokine storm’ [12,13]. Thus, anticoagulation therapy is often utilised for COVID-19 patients, either to prevent thrombosis, or to treat arising thrombosis in some patients, including PE and DVT [13,14,15,16,17,18,19].

We were also intrigued to learn of some reports in some geographic jurisdictions that identified a shift in anticoagulant usage during COVID-19 outbreaks [20,21,22], potentially away from VKAs. These changes were suggested as also potentially increasing during ‘COVID-19 associated lockdown periods’, where clinical visits to caring physicians or to pathology collection sites for INR monitoring became increasingly difficult, or where changes were suggested according to expert guidance. We therefore decided to evaluate for evidence of such shifts for Australia.

## 2. Materials and Methods

We used a similar approach to our earlier reports [7,8]. Data on anticoagulant availability were derived from the freely accessible pharmaceutical benefits scheme (PBS) online portal (https://www.pbs.gov.au/pbs/home, accessed on 5 February 2024) [23]. Under the PBS, the Australian government subsidises the cost of medicine for most medical conditions and lists all of the medicines available to be dispensed to patients at a government-subsidised price. Some PBS-listed medicines can only be prescribed for specific therapeutic uses. For example, while dabigatran is subsidised for prevention of stroke or systemic embolisation in non-valvular AF, it is not subsidised for treatment of VTE, even though this may be an approved indication. Searches were performed for the oral anticoagulants available in Australia (warfarin, dabigatran, rivaroxaban, apixaban) to identify the different formulations available. Subsequently, additional data on anticoagulant use and cost were available for each drug from the Medicare statistics website (http://medicarestatistics.humanservices.gov.au/statistics/pbs_item.jsp, accessed on 5 February 2024) [24]. Data can be downloaded for services or benefit by year and by state, as well as other categories.

Data on COVID-19 were obtained using the National Communicable Disease Surveillance Dashboard available at the National Notifiable Disease Surveillance System (https://nindss.health.gov.au/pbi-dashboard/, accessed on 5 February 2024) [25]. Again, data are available by year and by Australian state, with additional data available by year quarter.

To see if patterns for OAT usage were influenced by population changes, data were also obtained for Australian population changes from the Australian Bureau of Statistics (https://www.abs.gov.au/statistics/people/population/national-state-and-territory-population/latest-release, accessed on 5 February 2024) [26].

## 3. Results

Table 1 provides a summary of the oral anticoagulants currently available in Australia, inclusive of available brands, and the related PBS codes. Warfarin is the only VKA available in Australia (although available as two different brands: Coumadin, Marevan). Dabigatran (etexilate) was originally released as the brand Pradaxa, but several generics have since been released. Rivaroxaban was originally released as the brand Xarelto, but several generics have also since been released. Apixaban is currently only available as the original released brand Eliquis. Each OAC may be prescribed in different doses and quantities, for different applications or clinical indications.

**Figure 1:**
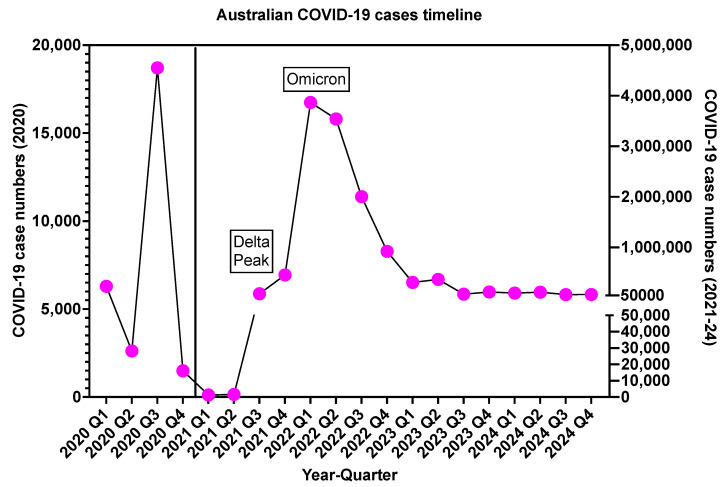
Data for COVID-19 cases according to year and quarter. There were several peaks associated with COVID-19 lockdowns in 2020 and 2021/2022. The 2021/2022 peaks were orders of magnitude higher than the 2020 peak. The pink dots indicate reported case numbers, and the solid black line indicates the trend line.

Data on Australian COVID-19 cases during the pandemic/endemic are shown in Figure 1. The first case of COVID-19 in Australia occurred in early 2020, with a peak outbreak in quarter 3 (Q3) of 2020, with this also representing the first COVID-19 lockdown in various Australia states. Cases of COVID-19 settled down briefly until mid-2021. Subsequently, the emergence of several disease variants resulted in additional COVID-19 peaks (for example for the Delta and Omicron variants in late 2021 and in 2022, respectively). This again led to additional COVID-19 lockdowns in various Australia states. Of interest, the peaks for the Delta and Omicron variants were magnitudes higher than the initial 2020 peak. As the variants continued to evolve, the number of reported COVID-19 cases began to achieve a sort of steady state. Accordingly, Australia, along with the rest of the world, accepted COVID-19 as an endemic disease. It should also be noted that the emergence of point-of-care testing, the broad diffusion of self-testing and lateral flow immunoassays for COVID-19 replaced the earlier genetic testing. Thus, the requirement to report COVID-19 only when genetically confirmed would suggest that these documented reported numbers of COVID-19 greatly underestimate current COVID-19 prevalence. Nevertheless, although emerging variants appear to be even more infectious than previous strains, they may lead to less severe disease.

**Figure 2:**
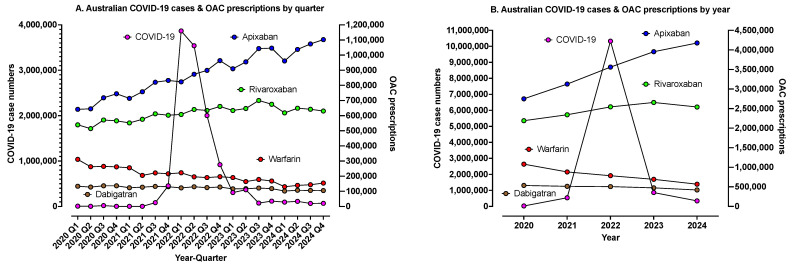
Usage of oral anticoagulants over the COVID-19 period. (**A**) Data separated into yearly quarters, with superimposed data on oral anticoagulant (OAC) PBS prescription data. There were no obvious signals for changed prescription/usage according to COVID-19 peaks/changes. (**B**) Data as per Figure 2A but only showing data for each year.

Usage of oral anticoagulants over the COVID-19 period is shown in Figure 2A,B. There was a general trend over this period to increasing use of apixaban and rivaroxaban and decreasing use of warfarin and dabigatran. However, we cannot identify any obvious signal of change related to COVID-19 peaks or lockdowns.

**Figure 3:**
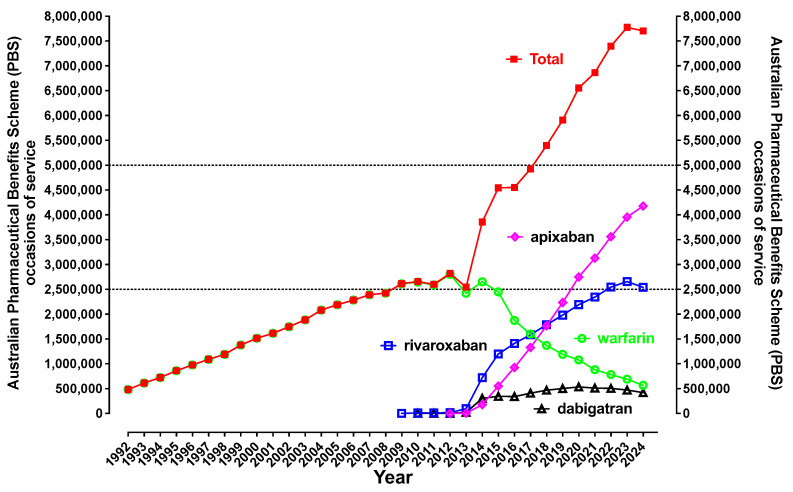
Occasions of service for oral anticoagulant (OAC) usage in Australia, 1992–2024 inclusive. Number of ‘occasions of service’ relate to prescriptions reimbursed by the Pharmaceutical Benefits Scheme (PBS) for various oral anticoagulants in Australia during 1992–2024 inclusive. There was a doubling of prescriptions for these anticoagulant drugs from 2012 (when current DOACs emerged) to 2016 (i.e., a 4-year period), this representing an increase equivalent to that experienced in the preceding 15-year period. A near doubling also occurred from 2016 to 2024.

Figure 3 shows the trend for oral anticoagulant usage over the period of 1992 to 2024. Warfarin was the only oral anticoagulant available in Australia until 2009, when rivaroxaban was released, followed by dabigatran in 2010 and apixaban in 2012. The initial growth in dabigatran usage was largely as a replacement for warfarin for prevention of AF-associated thromboembolic stroke, but usage numbers started to decline in 2020. Use of both rivaroxaban and apixaban increased from 2013 and continue to rise. However, apixaban is now ‘king’ of the oral anticoagulants in Australia, overtaking rivaroxaban in 2019. Nevertheless, the increase in the total oral anticoagulant usage over time suggests that these agents may no longer just be replacing warfarin, but likely also being increasingly prescribed for prior oral anticoagulant naïve patients. As mentioned earlier, this ‘extra burden of anticoagulation’ is likely to include all orthopedic indications for preventing VTE, plus chronic stable atherosclerotic disease (rivaroxaban). Furthermore, patients with VTE who used to be treated with a limited course of warfarin for 3–6 months on average are more likely to remain on indefinite therapy on a DOAC. Finally, the burden of regular monitoring and dose adjustments may have caused some clinicians to not prescribe warfarin for select patients, with this no longer being a concern for the DOACs.

**Figure 4:**
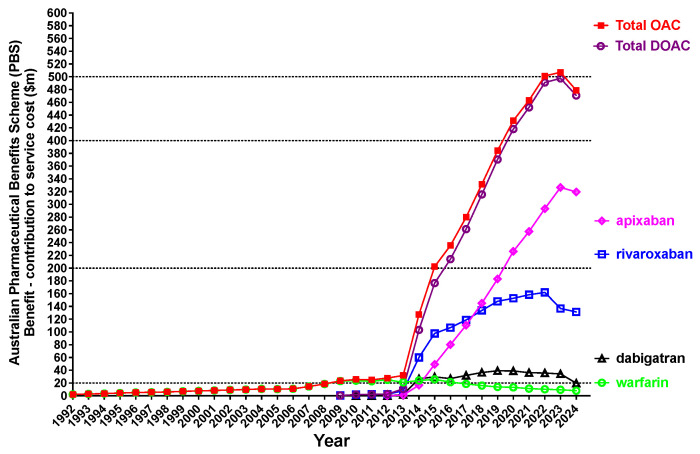
Associated costs for oral anticoagulant (OAC) usage in Australia, 1992–2024 inclusive (i.e., service cost of items identified in Figure 3. There has been an almost 25-fold increase in costs for these prescriptions since 2013 (i.e., last 11 years), and reaching half a billion dollars in 2023.

Figure 4 shows the cost of these PBS services. Warfarin is the cheapest of the available OACs, and also considering less usage, the cost of both warfarin and dabigatran are overshadowed by the costs associated with rivaroxaban and apixaban. Of interest, rivaroxaban costs seem to have decreased in 2023 compared to 2022, despite the growth in usage (Figure 3). This may be related to the release and probable increased use of cheaper rivaroxaban generics (Table 1 and Table 2). Nevertheless, the total cost of oral anticoagulant drugs reached a plateau of half a billion dollars in 2023. Finally, 2024 saw the first overall decrease in costs, along with reduced overall costs for apixaban, perhaps also reflecting PBS pricing adjustments for certain PBS codes (Table 2).

**Figure 5:**
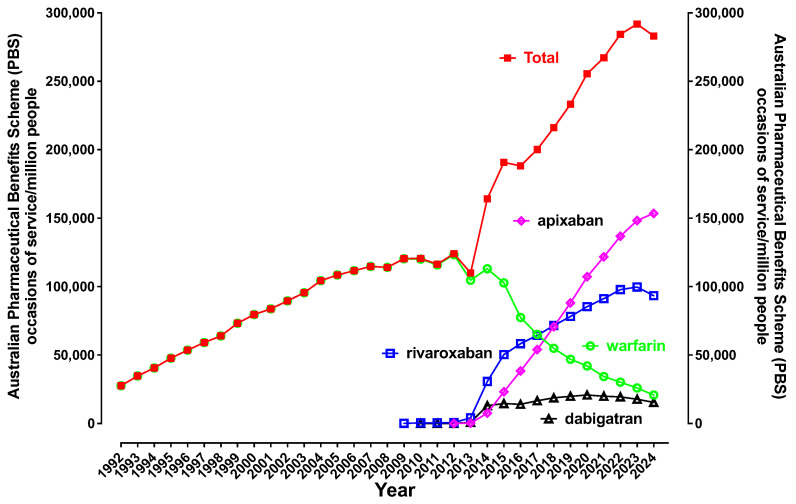
Occasions of service for oral anticoagulant (OAC) usage in Australia, 1992–2024 inclusive, shown per million people to adjust for population changes.

Given the increasing usage patterns in Figure 3, we wanted to make sure this was not just due to an increasing population, for example due to increased migration, although migration rates would have been low over the early years of COVID-19. Thus, Figure 5 provides oral anticoagulant usage data adjusted by population. The pattern is similar to that shown in Figure 3.

## 4. Discussion

Anticoagulants may be prescribed to patients who have suffered a thromboembolic event such as a DVT or PE, or who are at risk of a thromboembolic event (e.g., from AF, or hip/knee surgery) [1,2,3,27]. For those in the community setting, the most commonly utilised anticoagulants, for both convenience and safety/efficacy, are oral anticoagulants, with these currently comprising either VKAs or DOACs. The landscape of oral anticoagulant use and availability is ever evolving. In Australia, warfarin represents the historical or classical drug, and was the only oral anticoagulant available until 2009, with the emergence of DOACs, initially rivaroxaban and dabigatran, and later apixaban [7,8]. Although other DOACs are available internationally (e.g., edoxaban, betrixaban), these are not (yet) marketed in Australia.

In this report, we have updated oral anticoagulant use in Australia, using PBS data, and we show an ongoing increasing uptake of the anti-Xa DOAC agents (rivaroxaban, apixaban). We also show reduction in usage of the DOAC agent dabigatran (an anti-IIa agent) and the VKA warfarin (Figure 3 and Figure 5). Given increasing usage of oral anticoagulants in general, this seems to suggest increasing deployment due to the enhanced prevalence of some medical conditions like AF, in addition to just replacement of warfarin and dabigatran. To support the situation for AF, a recent study of anticoagulation for AF in the Australian primary care setting over 10 years reported an increase in the proportion prescribed an oral anticoagulant, which increased from 39.5% (95% CI 38.6–40.5%) in 2009 to 52.0% (95% CI 51.5–52.4%) in 2018 (*p*-value for trend < 0.001) [28]. Another study from the US reported on anticoagulant patterns for AF patients with an elevated stroke risk from 2011 to 2020 [29]. The authors reported that overall anticoagulation rates increased from 56.3% to 64.7%, as DOAC use increased steadily (from 4.7% to 47.9%), while warfarin use declined (from 52.4% to 17.7%). We also note evidence that detection rates for AF are also increasing globally [11]. Another study, from Italy, reported on continued rivaroxaban use in AF with ablation [30].

We also show in the current report the increasing cost of oral anticoagulant usage, with this reaching a half billion dollars in 2023. However, costs for some agents (e.g., rivaroxaban) may begin to decrease, despite increased usage, due to the release and increasing use of generics as well as adjusted PBS pricing (Figure 4; Table 2). There are no generics available for apixaban in Australia as yet, but pricing has on balance also fallen (Table 2), and apixaban generics will be expected to be released soon.

Despite some previous literature suggesting that COVID-19 surges/lockdowns have had an effect of oral anticoagulant usage trends [20,21,22], Australian data did not support this as a local outcome (Figure 2). In theory, COVID-19 increases the risk of thrombosis [13,14,15,16,17,18,19], and may also prevent patients from attending clinical appointments or pathology collection sites for INR monitoring. Further, certain types of COVID-19 vaccines may also pose a risk for thrombosis [31,32,33]. In particular, global outbreaks of vaccine-induced thrombocytopenia with thrombosis (VITT) or thrombotic thrombocytopenia syndrome (TTS) have been reported following immunisation with adenovirus-based vaccines, with Australian cases peaking in 2021, with over 1000 patients evaluated [34]. Naturally, the most severe cases of both COVID-19 and VITT/HIT would be managed within the hospital setting, and thus any associate anticoagulant data would not be captured in the current evaluation. However, once such patients are released into the community, and transitioned to oral anticoagulant therapy, these will be recorded and analysed in the ongoing analysis. In particular, the Australian study identified that many VITT patients suffered from classical thrombotic events, such as PE/DVT, and thus would have been managed primarily in the community after hospital discharge [34].

Although VKAs reflect cheap and clinically effective oral anticoagulants, they have slow onset of action, higher risk of bleeding, large inter-individual variation in anticoagulant response and many food and drug interactions [35,36,37]. Such limitations have driven advances in oral anticoagulant therapy, including development and marketing of DOACs, which have fewer limitations and similar or improved safety/efficacy. Several other oral anticoagulants are in development [4,5,6,38]. Clinical indications for DOACs and other oral anticoagulants in development are similar to one another and also to VKAs, and thus these will replace VKAs over time. However, approved and/or publicly subsidised indications geographically differ, depending on local regulatory approvals/clearances. Subsidised indications in Australia are given in Table 1. These indications align to, but do not comprise all approved indications. Although all DOACs have proven efficacy and safety, there are conditions for which their efficacy has not (yet) been shown or where concerns exist (e.g., patient groups such as obese, triple positive antiphospholipid syndrome, mechanical valves). DOACs are marketed as not requiring regular laboratory monitoring, thus being more convenient than classical anticoagulants. However, testing for DOACs is available, and sometimes performed to monitor patient compliance or assess for drug accumulation, or as a presurgical screen [9,39]. On the other hand, as pharmaceutical drugs, DOACs are more expensive than VKAs (Table 2). Conversely, this may be offset by the reduced need for monitoring (i.e., INRs for VKAs) and reduced clinical visits for INR testing and warfarin dose adjustments.

Our data have several limitations and do not fully inform on total drug use or patient numbers. First, these agents may be prescribed in multiple formulations (Table 1). For example, warfarin is available as two different drugs and four different formulations. Apixaban, dabigatran and rivaroxaban are also available in several different formulations, as well as potentially different drug versions given recent and ongoing release of generics. Second, the data restriction to PBS means that data on anticoagulant use related to private insurance claims, hospital prescriptions and out-of-pocket medication purchases are not included in the analysis. This will result in an incomplete representation of oral anticoagulation therapy usage.

Finally, for this report, it is important to recognise that there are several risks associated with the use of any anticoagulant. These comprise both clinical risks and also those related to patient misdiagnosis when pathology tests are performed on anticoagulant-treated patients [40,41,42]. Despite high safety profiles, all anticoagulants carry an unavoidable risk of bleeding. For VKAs, regular laboratory and clinical monitoring acts to safeguard against drug accumulation (due to compliance failure or drug/food interactions). The most often-used test is the routine INR, although alternate assays are available in some localities [43]. If required, reversal can be achieved using vitamin K (oral or parental) and/or plasma products (including prothrombin complex concentrate [PCC]). Bleeding due to DOAC drug accumulation is also possible. Here, reduction in clinical contact and lack of regular laboratory monitoring may not identify drug accumulation until suggested by breakthrough bleeding. DOACs are variably metabolised or cleared through liver and renal mechanisms. Therefore, monitoring of liver and renal function may be important considerations, particularly in the (especially acutely) ill and elderly, and evidence is accumulating that such cases may warrant drug dose reductions. If required, reversal can be achieved using specific antidotes (e.g., idarucizumab for dabigatran [44]; andexanet alfa for apixaban and rivaroxaban [45]) and/or plasma products (e.g., PCC). Conversely, patients may suffer thrombotic events while on OAT. If poor compliance is suggested, drug testing may inform on plasma levels, and whether these are within expected therapy ranges [9,39]. Consideration can also be given to shift the anticoagulant, based on concepts of personalised medicine.

## 5. Conclusions

Although all currently available anticoagulant drugs have advantages and limitations, DOAC usage is constantly increasing and gradually offsetting the use of other agents including heparin and VKAs for many clinical indications. Nevertheless, patients and clinicians need to become fully appraised of these newer drugs to maximise benefits and limit drawbacks. Clinicians should also be informed that the OAT landscape continues to change, given emerging agents such as the oral anti-XI agents (e.g., asundexian, milvexian) [4,5,6].

## Figures and Tables

**Table 1:** Current PBS oral anticoagulant Codes and indications.

Oral Anticoagulant, Available Brands and PBS Codes	Presentation (Dose, Pack Size)	Clinical Indication(s)
***Warfarin sodium (Coumadin, Marevan)***		
2843P	1 mg tablet, 50	Prevention of stroke or systemic embolism
2209G	2 mg tablet, 50	Continuing treatment of DVT/PE
2844Q	3 mg tablet, 50	Prevention of recurrent VTE
2211J	5 mg tablet, 50	Coronary occlusion adjunctive treatment
***Dabigatran etexilate (Pradaxa; newer generics: ARX-Dabigatran, Dabigatran Sandoz, PHARMACOR DABIGATRAN)***		
9318K	75 mg capsule, 10	Prevention of VTE after total hip replacement
9322P	75 mg capsule, 10	Prevention of VTE after total knee replacement
9319L	110 mg capsule, 10	Prevention of VTE after total hip replacement
9323Q	110 mg capsule, 10	Prevention of VTE after total knee replacement
13489Y, 2769R, 13523R, 2753X	150 mg capsule, 60	Prevention of stroke or systemic embolism
9321N	110 mg capsule, 60	Prevention of VTE after total hip replacement
9320M	75 mg capsule, 60	Prevention of VTE after total hip replacement
***Rivaroxaban (Xarelto; newer generics: RIVOXA, Rivaroxaban-Teva, iXarola, Rivaroxaban Sandoz)***		
12192Q, 12197Y, 13366L	2.5 mg tablet, 60	Chronic stable atherosclerotic disease
11633G, 13521P	10 mg tablet, 30	Prevention of recurrent VTE
9467G	10 mg tablet, 30	Prevention of VTE after total hip replacement
9466F	10 mg tablet, 15	Prevention of VTE after hip replacement
9469J	10 mg tablet, 15	Prevention of VTE after knee replacement
2160Q	15 mg tablet, 42	Initial treatment of DVT/PE
13462M, 2268J	20 mg tablet, 28	Continuing treatment of DVT/PE; prevention of recurrent VTE, Prevention of stroke or systemic embolism
13463N, 2691P	15 mg tablet, 28	Prevention of stroke or systemic embolism
***Apixaban (Eliquis)***		
13525W	5 mg tablet, 60	Prevention of stroke or systemic embolism
2735Y	5 mg tablet, 60	Continuing treatment of DVT/PE; prevention of stroke or systemic embolism
5500L	2.5 mg tablet, 20	Prevention of VTE after total knee or hip replacement
5054B	2.5 mg tablet, 30	Prevention of VTE after total knee replacement
13464P	2.5 mg tablet, 60	Prevention of stroke or systemic embolism
2744K	2.5 mg tablet, 60	Prevention of recurrent VTE; prevention of stroke or systemic embolism
5061J	2.5 mg tablet, 60	Prevention of VTE after total hip replacement
10414D	5 mg tablet, 28	Initial treatment of DVT/PE

DVT, deep vein thrombosis; PE, pulmonary thrombosis; VTE, venous thromboembolism.

**Table 2:** PBS oral anticoagulant pricing ^1^.

Oral Anticoagulant, PBS Codes/Generics	Pricing in 2020	Pricing in 2024 (Original Item)	Pricing in 2024 (Generic Items)	Comment (2024 vs. 2020)
***Warfarin sodium (Coumadin, Marevan)***				
2843P	16.16	16.16	NA	No change
2209G	16.35	16.35	NA	No change
2844Q	16.42	16.42	NA	No change
2211J	16.86	16.86	NA	No change
***Dabigatran etexilate (Pradaxa; newer generics: ARX-Dabigatran, Dabigatran Sandoz, PHARMACOR DABIGATRAN)***				
9318K	44.39	31.25	NA	Reduced
9322P	NA	35.21	NA	NA
9319L	37.25	26.31	NA	Reduced
9323Q	NA	26.31	NA	NA
13489Y	NA	102.17	84.31	Generics cheaper
2769R	88.77	57.81	48.88	Reduced, generics cheaper
13523R	NA	102.69	84.83	Generics cheaper
2753X	88.77	58.07	49.14	Reduced, generics cheaper
9321N	NA	58.07	49.14	Generics cheaper
9320M	110.18	79.63	79.63	Reduced
***Rivaroxaban (Xarelto; newer generics: RIVOXA, Rivaroxaban-Teva, iXarola, Rivaroxaban Sandoz)***				
12192Q, 12197Y	NA	60.12	60.12	NA
13366L	NA	84.15	84.15	NA
11633G	92.99	66.36	66.36	Reduced
13521P	NA	93.55	93.55	NA
9467G	NA	53.50	53.50	NA
9466F	52.24	39.68	39.68	Reduced
9469J	NA	39.68	39.68	NA
2160Q	125.6	86.85	86.85	Reduced
13462M	NA	110.23	110.23	NA
2268J	87.56	61.61	61.61	Reduced
13463N	NA	111.45	111.45	NA
2691P	87.56	62.22	62.22	Reduced
***Apixaban (Eliquis)***				
13525W	NA	171.25	NA	NA
2735Y	93.31	93.73	NA	Small increase
5500L	38.76	38.90	NA	Small increase
5054B	52.4	51.86	NA	Small reduction
13464P	NA	171.25	NA	NA
2744K	93.31	90.73	NA	Reduced
5061J	NA	69.13	NA	NA
10414D	49.67	49.27	NA	Small reduction

^1^ dispensed price for maximum quantity (Australian $). NA, not applicable or not available.

## Data Availability

Data supporting results reported in this manuscript can be found in the public domain, at the websites identified in the reference list.

## References

[1] Hirsh J., de Vries T.A.C., Eikelboom J.W., Bhagirath V., Chan N.C. (2023). Clinical Studies with Anticoagulants that Have Changed Clinical Practice. Semin. Thromb. Hemost..

[2] Lippi G., Mattiuzzi C., Favaloro E.J. (2023). Letter to the Editor: 10-Year Evolution in Worldwide Usage of Anticoagulant Drugs. Semin. Thromb. Hemost..

[3] Lippi G., Gosselin R., Favaloro E.J. (2019). Current and Emerging Direct Oral Anticoagulants: State-of-the-Art. Semin. Thromb. Hemost..

[4] Piccini J.P., Patel M.R., Steffel J., Ferdinand K., Van Gelder I.C., Russo A.M., Ma C.S., Goodman S.G., Oldgren J., Hammett C. (2025). Asundexian versus Apixaban in Patients with Atrial Fibrillation. N. Engl. J. Med..

[5] Barnes G.D. (2024). New targets for antithrombotic medications: Seeking to decouple thrombosis from hemostasis. J. Thromb. Haemost..

[6] Weitz J.I., Strony J., Ageno W., Gailani D., Hylek E.M., Lassen M.R., Mahaffey K.W., Notani R.S., Roberts R., Segers A. (2021). Milvexian for the Prevention of Venous Thromboembolism. N. Engl. J. Med..

[7] Favaloro E.J., Pasalic L., Lippi G. (2017). Replacing warfarin therapy with the newer direct oral anticoagulants, or simply a growth in anticoagulation therapy? Implications for Pathology testing. Pathology.

[8] Favaloro E.J., Pasalic L., Lippi G. (2020). Oral anticoagulation therapy: An update on usage, costs and associated risks. Pathology.

[9] Douxfils J., Adcock D.M., Bates S.M., Favaloro E.J., Gouin-Thibault I., Guillermo C., Kawai Y., Lindhoff-Last E., Kitchen S., Gosselin R.C. (2021). 2021 Update of the International Council for Standardization in Haematology Recommendations for Laboratory Measurement of Direct Oral Anticoagulants. Thromb. Haemost..

[10] Favaloro E.J., Arunachalam S., Chapman K., Pasalic L. (2025). Continued Harmonization of the International Normalized Ratio (INR) across a large laboratory network: Evidence of sustained low inter-laboratory variation and bias after a change in instrumentation. Am. J. Clin. Pathol..

[11] Lippi G., Sanchis-Gomar F., Cervellin G. (2021). Global epidemiology of atrial fibrillation: An increasing epidemic and public health challenge. Int. J. Stroke.

[12] Lippi G., Sanchis-Gomar F., Favaloro E.J., Lavie C.J., Henry B.M. (2021). Coronavirus Disease 2019-Associated Coagulopathy. Mayo Clin. Proc..

[13] Bikdeli B., Madhavan M.V., Jimenez D., Chuich T., Dreyfus I., Driggin E., Nigoghossian C.D., Ageno W., Madjid M., Guo Y. (2020). COVID-19 and Thrombotic or Thromboembolic Disease: Implications for Prevention, Antithrombotic Therapy, and Follow-up. JACC State-of-the-Art Review. J. Am. Coll. Cardiol..

[14] Bikdeli B., Madhavan M.V., Gupta A., Jimenez D., Burton J.R., Der Nigoghossian C., Chuich T., Nouri S.N., Dreyfus I., Driggin E. (2020). Pharmacological Agents Targeting Thromboinflammation in COVID-19: Review and Implications for Future Research. Thromb. Haemost..

[15] Hashemi A., Madhavan M.V., Bikdeli B. (2020). Pharmacotherapy for Prevention and Management of Thrombosis in COVID-19. Semin. Thromb. Hemost..

[16] Ortega-Paz L., Talasaz A.H., Sadeghipour P., Potpara T.S., Aronow H.D., Jara-Palomares L., Sholzberg M., Angiolillo D.J., Lip G.Y.H., Bikdeli B. (2023). COVID-19-Associated Pulmonary Embolism: Review of the Pathophysiology, Epidemiology, Prevention, Diagnosis, and Treatment. Semin. Thromb. Hemost..

[17] Rizk J.G., Gupta A., Lazo J.G., Sardar P., Henry B.M., Lavie C.J., Effron M.B. (2023). To Anticoagulate or Not to Anticoagulate in COVID-19: Lessons after 2 Years. Semin. Thromb. Hemost..

[18] Candeloro M., Schulman S. (2023). Arterial Thrombotic Events in Hospitalized COVID-19 Patients: A Short Review and Meta-Analysis. Semin. Thromb. Hemost..

[19] Di Minno A., Ambrosino P., Calcaterra I., Di Minno M.N.D. (2020). COVID-19 and Venous Thromboembolism: A Meta-analysis of Literature Studies. Semin. Thromb. Hemost..

[20] Alkhameys S., Barrett R. (2022). Impact of the COVID-19 pandemic on England’s national prescriptions of oral vitamin K antagonist (VKA) and direct-acting oral anticoagulants (DOACs): An interrupted time series analysis (January 2019–February 2021). Curr. Med. Res. Opin..

[21] Curtis H.J., MacKenna B., Walker A.J., Croker R., Mehrkar A., Morton C., Bacon S., Hickman G., Inglesby P., Bates C. (2021). OpenSAFELY: Impact of national guidance on switching anticoagulant therapy during COVID-19 pandemic. Open Heart.

[22] Dale C.E., Takhar R., Carragher R., Katsoulis M., Torabi F., Duffield S., Kent S., Mueller T., Kurdi A., Le Anh T.N. (2023). The impact of the COVID-19 pandemic on cardiovascular disease prevention and management. Nat. Med..

[23] Pharmaceutical Benefits Scheme (PBS). https://www.pbs.gov.au/pbs/home.

[24] Pharmaceutical Benefits Schedule Item Reports. http://medicarestatistics.humanservices.gov.au/statistics/pbs_item.jsp.

[25] National Notifiable Disease Surveillance System. https://nindss.health.gov.au/pbi-dashboard/.

[26] Australian Bureau of Statistics. https://www.abs.gov.au/statistics/people/population/national-state-and-territory-population/latest-release.

[27] Ageno W., Gallus A.S., Wittkowsky A., Crowther M., Hylek E.M., Palareti G. (2012). Oral anticoagulant therapy: Antithrombotic Therapy and Prevention of Thrombosis, 9th ed: American College of Chest Physicians Evidence-Based Clinical Practice Guidelines. Chest.

[28] Bezabhe W.M., Bereznicki L.R., Radford J., Wimmer B.C., Curtain C., Salahudeen M.S., Peterson G.M. (2021). Ten-Year Trends in the Use of Oral Anticoagulants in Australian General Practice Patients With Atrial Fibrillation. Front. Pharmacol..

[29] Navar A.M., Kolkailah A.A., Overton R., Shah N.P., Rousseau J.F., Flaker G.C., Pignone M.P., Peterson E.D. (2022). Trends in Oral Anticoagulant Use Among 436,864 Patients with Atrial Fibrillation in Community Practice, 2011 to 2020. J. Am. Heart Assoc..

[30] Lavalle C., Pierucci N., Mariani M.V., Piro A., Borrelli A., Grimaldi M., Rossillo A., Notarstefano P., Compagnucci P., Dello Russo A. (2024). Italian Registry in the Setting of Atrial Fibrillation Ablation with Rivaroxaban—IRIS. Minerva Cardiol. Angiol..

[31] Favaloro E.J., Pasalic L. (2021). COVID-19 vaccine induced (immune) thrombotic thrombocytopenia (VITT)/thrombosis with thrombocytopenia syndrome (TTS): An update. Aust. J. Med. Sci..

[32] Hafeez M.U., Ikram M., Shafiq Z., Sarfraz A., Sarfraz Z., Jaiswal V., Sarfraz M., Chérrez-Ojeda I. (2021). COVID-19 Vaccine-Associated Thrombosis with Thrombocytopenia Syndrome (TTS): A Systematic Review and Post Hoc Analysis. Clin. Appl. Thromb. Hemost..

[33] Selvadurai M.V., Favaloro E.J., Chen V.M. (2023). Mechanisms of Thrombosis in Heparin-Induced Thrombocytopenia and Vaccine-Induced Immune Thrombotic Thrombocytopenia. Semin. Thromb. Hemost..

[34] Favaloro E.J., Clifford J., Leitinger E., Parker M., Sung P., Chunilal S., Tran H., Kershaw G., Fu S., Passam F. (2022). Assessment of immunological anti-platelet factor 4 antibodies for vaccine-induced thrombotic thrombocytopenia (VITT) in a large Australian cohort: A multicentre study comprising 1284 patients. J. Thromb. Haemost..

[35] Lawal O.D., Aronow H.D., Shobayo F., Hume A.L., Taveira T.H., Matson K.L., Zhang Y., Wen X. (2023). Comparative Effectiveness and Safety of Direct Oral Anticoagulants and Warfarin in Patients with Atrial Fibrillation and Chronic Liver Disease: A Nationwide Cohort Study. Circulation.

[36] Talasaz A.H., McGonagle B., HajiQasemi M., Ghelichkhan Z.A., Sadeghipour P., Rashedi S., Cuker A., Lech T., Goldhaber S.Z., Jennings D.L. (2024). Pharmacokinetic and Pharmacodynamic Interactions between Food or Herbal Products and Oral Anticoagulants: Evidence Review, Practical Recommendations, and Knowledge Gaps. Semin. Thromb. Hemost..

[37] Mar P.L., Gopinathannair R., Gengler B.E., Chung M.K., Perez A., Dukes J., Ezekowitz M.D., Lakkireddy D., Lip G.Y.H., Miletello M. (2022). Drug Interactions Affecting Oral Anticoagulant Use. Circ. Arrhythmia Electrophysiol..

[38] Fredenburgh J.C., Weitz J.I. (2021). New anticoagulants: Moving beyond the direct oral anticoagulants. J. Thromb. Haemost..

[39] Lippi G., Favaloro E.J. (2024). Pearls and Pitfalls in the Measurement of Direct Oral Anticoagulants. Semin. Thromb. Hemost..

[40] Favaloro E.J., Mohammed S., Curnow J., Pasalic L. (2019). Laboratory testing for lupus anticoagulant (LA) in patients taking direct oral anticoagulants (DOACs): Potential for false positives and false negatives. Pathology.

[41] Favaloro E.J. (2019). Danger of false negative (exclusion) or false positive (diagnosis) for ‘congenital thrombophilia’ in the age of anticoagulants. Clin. Chem. Lab. Med..

[42] Favaloro E.J., Lippi G. (2017). Interference of direct oral anticoagulants in haemostasis assays: High potential for diagnostic false positives and false negatives. Blood Transfus..

[43] Favaloro E.J., Pasalic L. (2024). Innovative Diagnostic Solutions in Hemostasis. Diagnostics.

[44] Brennan Y., Favaloro E.J., Pasalic L., Keenan H., Curnow J. (2019). Lessons learnt from local real-life experience with idarucizumab for the reversal of dabigatran. Intern. Med. J..

[45] Kalathottukaren M.T., Creagh A.L., Abbina S., Lu G., Karbarz M.J., Pandey A., Conley P.B., Kizhakkedathu J.N., Haynes C. (2018). Comparison of reversal activity and mechanism of action of UHRA, andexanet, and PER977 on heparin and oral FXa inhibitors. Blood Adv..

